# Oral Tolerance and Pyruvate Dehydrogenase in Patients with Primary Biliary Cirrhosis

**DOI:** 10.1080/1044667021000098561

**Published:** 2002-06

**Authors:** Ayako Suzuki, Judy van de Water, M. Eric Gershwin, Roberta Jorgensen, Paul Angulo, Keith Lindor

**Affiliations:** 1 Division of Gastroenterology and Hepatology Mayo Clinic W19A 200 First Street SW Rochester MN 55905 USA; 2 School of Health Science Kanazawa University Kanazawa Ishikawa Japan; 3 School of Medicine University of California—Davis Davis CA USA

## Abstract

Primary biliary cirrhosis (PBC) is a chronic cholestatic liver disease characterized by the immunological destruction of intralobular bile ducts and serum anti-mitochondrial antibodies (AMA). Based upon previous work of oral tolerance and autoimmunity, we hypothesized that feeding the mitochondrial autoantigens of PBC would alter the clinical course and the level of antimitochondrial antibodies. The bovine pyruvate dehydrogenase complex (PDC) was purified and 5 mg fed in gelatin capsules to 6 patients with early stage PBC for 6 months. Antimitochondrial antibodies and liver biochemistries were measured at every 3 months for 12 months. The clinical trial was completed for all patients except for 1 who showed deterioration of pre-existing skin rash during treatment, which disappeared within 2 weeks after treatment was discontinued. However, after 1 year, neither the titers of AMAs nor liver biochemistries were significantly changed by this treatment. This is the first trial to test the efficacy of oral tolerance induction in PBC. However, the data, which limited in scope, did not demonstrate efficacy and further highlights the difficulties in showing continuing evidence of tolerance induction in autoimmunity.


*Developmental Immunology*, 2002 Vol. 9 (2), pp. 55–61

Taylor & Francis healthsciences

### Oral Tolerance and Pyruvate Dehydrogenase in Patients with Primary Biliary Cirrhosis

AYAKO SUZUKI[a,b], JUDY VAN De WATER[c], M. ERIC GERSHWIN[c], ROBERTA JORGENSEN[a], PAUL ANGULO[a] and KEITH LINDOR[a,*]

[a]*Division of Gastroenterology and Hepatology, Mayo Clinic, W19A, 200 First Street, SW, Rochester, MN 55905, USA;* [b]*School of Health Science, Kanazawa University, Kanazawa, Ishikawa, Japan;* [c]*School of Medicine, University of California—Davis, Davis, CA, USA*

Primary biliary cirrhosis (PBC) is a chronic cholestatic liver disease characterized by the immunological destruction of intralobular bile ducts and serum anti-mitochondrial antibodies (AMA). Based upon previous work of oral tolerance and autoimmunity, we hypothesized that feeding the mitochondrial autoantigens of PBC would alter the clinical course and the level of antimitochondrial antibodies. The bovine pyruvate dehydrogenase complex (PDC) was purified and 5 mg fed in gelatin capsules to 6 patients with early stage PBC for 6 months. Antimitochondrial antibodies and liver biochemistries were measured at every 3 months for 12 months. The clinical trial was completed for all patients except for 1 who showed deterioration of pre-existing skin rash during treatment, which disappeared within 2 weeks after treatment was discontinued. However, after 1 year, neither the titers of AMAs nor liver biochemistries were significantly changed by this treatment. This is the first trial to test the efficacy of oral tolerance induction in PBC. However, the data, which limited in scope, did not demonstrate efficacy and further highlights the difficulties in showing continuing evidence of tolerance induction in autoimmunity.

*Keywords*: Antimitochondrial antibodies; Autoimmunity; Oral tolerance; Pyruvate dehydrogenase

### INTRODUCTION

The current treatment of primary biliary cirrhosis (PBC) is less than fully satisfactory. In spite of the disease's autoimmune nature, most immunosuppressive agents, including corticosteroids (Mitchison *et al.*, 1989), cyclosporin (Wiesner *et al.*, 1990; Lombard *et al.*, 1993), azathioprine (Heathcote *et al.*, 1976), and methotrexate (Kaplan and Knox, 1991) fail to show satisfactory clinical benefit. Ursodeoxycholic acid (UDCA) is a safe and effective medication for most patients and currently is the therapy of choice for patients with PBC (Lindor *et al.*, 1994). Treatment with UDCA leads to not only biochemical and symptomatic improvement but also reduces the risk of developing varices and improves survival free of liver transplantation (Lindor *et al.*, 1996; 1997; Poupon *et al.*, 1997). However, up to two thirds of patients with PBC have abnormal liver tests despite UDCA treatment (Angulo *et al.*, 1999). Several combination trials of UDCA with other agents, such as methotrexate (Lindor *et al.*, 1995), budesonide (Angulo *et al.*, 2000a), and silymarin (Angulo *et al.*, 2000b), have not resulted in evidence of benefit from these agents when added to UDCA.

PBC is characteristically associated with various autoimmune diseases and findings. One of the most common findings is positivity for serum anti-mitochondrial antibodies (AMA). In the late 1980s, the molecular targets of AMA were identified (Gershwin *et al.*, 1987) and promoted progress in understanding the pathophysiology of PBC. These antigens are molecules of the 2-oxoacid dehydrogenase complex located on the mammalian inner mitochondrial membrane, especially the E2 component of these enzymes (Van de Water *et al.*, 1988; Yeaman *et al.*, 1988). The enzyme complex includes pyruvate dehydrogenase complex (PDC), the branched chain α-ketoacid dehydrogenase complex (BCKD), and the oxoglutarate dehydrogenase complex (OGDC), E1, E2 and E3 (Yeaman, 1989). Among these molecular targets, AMA are most commonly directed to the E2 component of PDC (Leung *et al.*, 1992).

Oral tolerance is a potentially therapeutic form of immunological modulation and this approach of tolerance induction in autoimmune disease was first reported in experimental arthritis by oral administration of collagen type 2 (Nagler-Anderson *et al.*, 1986; Thompson and Staines, 1986). These reports encouraged attempts to test whether oral tolerance to the disease-specific autoantigens

*Corresponding author. Tel.: +1-507-284-1738. Fax: +1-507-266-4531. E-mail: lindor.keith@mayo.edu

ISSN 1044-6672 print © 2002 Taylor & Francis Ltd
DOI: 10.1080/1044667021000098561

was effective in other experimental autoimmune models for myasthenia gravis (Wang *et al.*, 1993), thyroiditis (Peterson and Braley-Mullen, 1995), encephalomyelitis (Bitar and Whitacre, 1988), and uveoretinitis (Nussenblatt *et al.*, 1990). There are also encouraging clinical data showing efficacy of oral tolerance in multiple sclerosis (Weiner *et al.*, 1993) and rheumatoid arthritis (Trentham *et al.*, 1993). These previous reports encouraged us to test our hypothesis by attempting to induce oral tolerance with the major autoantigens in PBC and PDC-E2.

### MATERIALS AND METHODS

### Subjects

Six patients with PBC were enrolled. The diagnosis of PBC was established based on the following criteria: (1) chronic cholestatic liver disease of at least 6 months' duration; (2) serum alkaline phosphatase level at least one and half times the upper limits of normal; (3) positive antimitochondrial antibody ($\geq$1:20 or $\geq$1.0 Units); (4) ultrasound, computed tomography (CT), or cholangiography of the biliary tree which excluded biliary obstruction; (5) recent liver biopsy (available for review) compatible with the diagnosis of PBC and showing stage 1 or 2 disease. Patients were excluded for the following criteria: (1) pregnancy; (2) anticipated need for transplantation in 1-year with less that 80% 1-year survival without liver transplantation as determined by the Mayo score; (3) other serious coexisting complications such as advanced malignancy or severe cardiopulmonary disease which would be expected to limit life expectancy to less than three years; (4) treatment with UDCA, methotrexate, corticosteroids, azathioprine, chlorambucil, cyclosporin, budesonide, d-penicillamine, or colchicine, in the preceding 3 months; (5) age less than 18 years of age or greater than 70 years of age; (6) findings highly suggestive of liver diseases of other etiology such as chronic alcoholic liver disease, chronic hepatitis B or C infection, autoimmune hepatitis, or sclerosing cholangitis; (7) patients unable to provide informed consent. The study was approved by the Mayo Institutional Review Board, and all patients gave informed consent for participation.

### Experimental Design

Six patients who met the criteria mentioned above were enrolled in the study. Before study entry, a complete medical history and physical examination were performed. The study duration was 12 months; for the first 6 months, each patient took daily study medication unless any adverse effects were reported; for the subsequent 6 months they were followed without any medication for PBC. Blood samples were obtained at baseline, and repeated at 3-month interval during the study duration. To test if these study medications induce oral tolerance, we measured autoantibodies including anti-PDC-E2, anti-BCKD-E2, and anti-OGDC-E2. We also measured liver biochemistries including serum alkaline phosphatase, serum aspartate aminotransferase, total bilirubin, and albumin to test if any improvement in these data were induced by oral administration of PDC.

### Material

PDC was isolated from beef heart using differential centrifugation. Fresh USDA inspected beef heart was obtained from a campus slaughterhouse and stored on ice in clean plastic bags. The beef heart was diced into small cubes and homogenized (1:2 w/w) in sterile 0.5 M sorbitol, 0.3 M sucrose, 0.1 mM EDTA and 50 mm Sodium phosphate at pH 7.4. Care was taken to keep the beef heart chilled. All materials including blender, knife, and centrifuge tubes used in this process were purchased exclusively for this project. The area used for preparation was cleaned thoroughly and lined with new clean, absorbent paper. The homogenate was centrifuged at 250*g* for 10 min, at 4°C. After filtering the supernatant through 4 layers of cheesecloth, this centrifugation was repeated to obtain clear supernatant. The clarified supernatant was decanted and centrifuged at 8000*g* for 10 min, at 4°C. The pellet was resuspended in chilled sorbitol buffer and respun at 8000*g* for 10 min. at 4°C. This step was then repeated. The mitochondria enriched pellet was resuspended in 0.02 M potassium phosphate, pH 6.5 to form a concentrated slurry. A 10 ml of this slurry was placed in a clean, chilled French press cell (which had been washed thoroughly and bathed in 70% ETOH for sterilization). The mitochondria were disrupted using 13,000 psi and the eluate was collected on ice. The membranes were removed by centrifugation at 20,000*g* for 30 min. at 4°C. The supernatant was collected and immediately frozen at −20°C. This solution was dialyzed against distilled water at 4°C and lyophylized. Protein content of the lyophylized material was measured using BioRad one step reagent and 10 μg loaded on 10% SDS page gel, transferred, and probed with anti-PDC-E2 (4Cb3) to verify the presence and integrity of PDC-E2. The final product contains enriched PDC, and includes other mitochondrial proteins such as BCKD and OGDC.

### Study Medication

The amount of PDC in the diet is estimated as about 0.0001–0.001% of total protein. In order to induce oral tolerance, we administered 5 mg PDC per day, which is 10–100 times more than the estimated amount in the regular diet. PDC was administered as a single gelatin capsule, taken once a night at least 2 h after the last meal, and continued for the first 6 months. For the subsequent 6 months, patients were tested every 3 months. During the 12-month study duration, no other medications for PBC such as UDCA were allowed. Patients receiving other treatment for PBC had been discontinued from those treatments at least 3 months before their enrolment.

TABLE I Clinical characteristics of enrolled patients at entry

| | Age (year) | Gender | Duration from Dx (months) | Serum AMA | Histologic stage |
|---|---|---|---|---|---|
| Patient 1 | 51 | F | 19 | 1:80 | 2 |
| Patient 2 | 63 | F | 9 | 1:80 | 2 |
| Patient 3 | 42 | F | 18 | 1:320 | 1 |
| Patient 4 | 44 | F | 15 | 1:20480 | 2 |
| Patient 5 | 48 | F | 0.5 | 1:80 | 1 |
| Patient 6 | 58 | F | 3 | 2.6* | 2 |

*Measured by ELISA, Level > 1.0 is considered positive.

### Autoantibody Analysis

Serum autoantibodies against PDC-E2, BCKD-E2, and OGDC-E2, were analyzed by a standard ELISA with serial two-fold dilutions of test sera, up to an $8 \times 10^{-6}$ dilution. One hundred microliter aliquot of patient serum mixed with 1% BSA/PBS was placed in each well of a PDC-E2-coated microtiter plate for 2 h at 37°C to bind to the plates. After binding, the plates were washed twice and incubated with peroxidase-conjugated, affinity-purified, polyvalent goat antihuman IgG. After washing, the plates were exposed to ABTS (2-azino-bis-thiosulfonate) substrate and then read at OD414 on a Multiscan machine (Victor 2 plate reader, Perkin Elmer, Boston, MA). The intra-assay variance for these analyses was between 0.05 and 0.082 standard error of the mean (SEM), while the inter-assay variance ranged between 0.06 and 1.1 SEM. A positive value was calculated as three standard deviation above the mean of the normal controls.

### Statistical Analysis

Results were expressed as mean values ± SEM. The Wilcoxon signed ranks test was used to compare baseline vs. 6 and 12 month values of autoantibodies and liver biochemistries.

### RESULTS

The clinical characteristics of the 6 enrolled patients at entry are summarized in Table I. All patients were female and the mean age was 51 ± 3 years (range 42–63). The mean duration from the first diagnosis made by liver biopsy to the study entry was 11 ± 3 months (range 0.5–19). All 6 patients were positive for anti-mitochondrial antibody. Histologic stage by liver biopsy was stage 1 in 2, and stage 2 in 4 patients. One patient who presented with a slight rash at entry discontinued the study medication on the 9th day since skin lesions deteriorated after the study medication was begun; the skin lesions improved within 2 weeks after the medication was discontinued. The remaining patients completed 6 months of study medication and the subsequent 6 months of observation. There were no adverse effects reported except for patient number 5 who noticed a mild deterioration of pre-existing general lymphadenopathy and Raynaud's phenomenon while receiving the study medication. These manifestations improved to the pre-study level after 6 months of medication.

The values at baseline for liver biochemistries and autoantibodies are shown in Table II. As shown in the table, serum ALP was above two times the upper limit of normal range for all patients and over five times for 1 patient. Serum AST was below two times the upper limit of normal range for all patients except for the same patient. Serum total bilirubin and albumin were normal for all patients. Autoantibodies directed to BCKD-E2, OGDC-E2, and PDC-E2 were positive for all patients with various levels among patients as shown, except for 1 patient whose data was not available.

Immunological data were available for 4 of 5 patients. At 6 months, none of the titers of anti BCKD-E2, OGDC-E2, and PDC-E2 antibodies differed from the baseline values ($p = 0.69$, 0.61, and 0.86, respectively). Likewise, at 12 months none of the titers of anti BCKD-E2, OGDC-E2, and PDC-E2 antibodies were different from baseline ($p = 0.30$, 0.57, and 0.45, respectively). With respect to individual data, the titers of anti-OGDC-E2 and PDC-E2 at 6 months showed an increase for patient No. 3 (Fig. 1). At 12 months, the titers of anti OGDC-E2 and PDC-E2

TABLE II The immunological data and liver biochemistries of patients at entry

| | Liver biochemistry | | | | Autoantibody (× 1000) | | |
|---|---|---|---|---|---|---|---|
| | ALP (U/l) | AST (U/l) | Total bilirubin (mg/dl) | Albumin (g/dl) | BC-E2 (O.D.) | OG-E2 (O.D.) | PDC-E2 (O.D.) |
| Patient 1 | 495 | 56 | 1.3 | 4.4 | 0.124 | 0.127 | 0.03 |
| Patient 2 | 467 | 38 | 0.4 | 3.8 | 0.132 | 0.059 | 0.001 |
| Patient 3 | 593 | 32 | 0.8 | 3.9 | 0.053 | 0.029 | 0.365 |
| Patient 4 | 1238 | 103 | 0.7 | 4.2 | – | – | – |
| Patient 5 | 466 | 31 | 0.4 | 4.2 | 0.098 | 0.131 | 0.293 |
| Mean ± SD | 652 ± 148 | 52 ± 14 | 0.7 ± 0.2 | 4.1 ± 0.1 | 0.102 ± 0.018 | 0.087 ± 0.025 | 0.172 ± 0.092 |
| Normal range | 81–213 | 12–31 | 0.1–1.1 | 3.5–5.0 | | | |

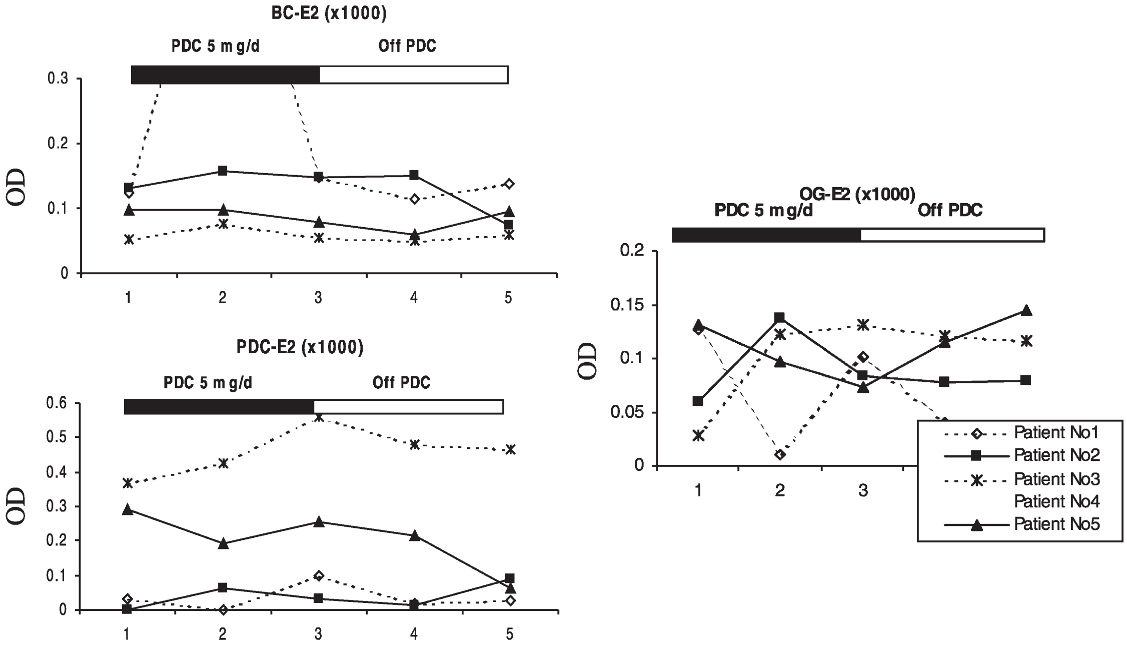

FIGURE 1 Anti-mitochondrial antibodies to three mitochondrial antigens, BCKD, OGDC, and PDC in patients before and after medication. (a) Anti-mitochondrial antibody to BCKD. (b) Anti-mitochondrial antibody to OGDC. (c) Anti-mitochondrial antibody to PDC. The horizontal axis of each chart is the time interval of 12 months study duration. The vertical axis is the titer of each antibody ( × 1000, O.D.). The dotted line with open diamonds represents patient No. 1. The solid line with closed squares represents patient No. 2. The dotted line with stars represents patient No. 3. The solid line with closed triangles represents patient No. 5.

antibody showed decreases for patients No. 1 and No. 5, respectively (Fig. 1). In contrast, the titers of anti-OGDC-E2 and PDC-E2 showed increases for patients No. 3, No. 2 and No. 3, respectively (Fig. 1). There was no consistency among the antibodies in terms of individual responses.

With respect to liver biochemistries, serum ALP, AST, total bilirubin, and albumin at 6-month were not different from baseline ($p = 0.94$, 0.90, 0.68, and 0.13, respectively). The values of serum ALP, AST, total bilirubin, and albumin at 12-month were also not different from the values at baseline ($p = 0.99$, 0.99, 0.96, and 0.16, respectively). No apparent improvement or deterioration was seen in terms of liver biochemistries for any of the patients during the study duration. Individual values were plotted in Fig. 2.

### DISCUSSION

Autoantibodies reactive to PDC-E2 are the most prevalent and disease-specific findings and are present in the serum of 95% of patients with PBC (Yeaman *et al.*, 1988). However, the role of AMA in the pathogenesis is not fully understood. There are lines of evidence supporting a pathophysiological significance of AMA. Immunohistochemical findings showing that the antibody-antigen complexes are located on the apical portion of the bile duct epithelial cells (Nakanuma *et al.*, 1995; Tsuneyama *et al.*, 1995; Leung *et al.*, 1997) suggest that AMA are not epiphenomenon of the disease but may play a significant role in the pathogenesis. Furthermore, mice sensitized intraperitoneally or subcutaneously with bovine PDC developed AMA and autoimmune cholangitis (Jones *et al.*, 2000). T cell and B cell reactivity to PDC-E2 in the early stage of the disease also support the idea that PDC-E2 has a significant role in the pathogenesis of PBC (Yeaman *et al.*, 1988; Jones *et al.*, 1995). This supportive evidence led us to hypothesize that immunological modulation in the early stage of the disease, designed to eliminate the titer of AMA, may improve the clinical course and hopefully provide a medication-free life to the patients.

In this trial, oral administration of bovine PDC was tested as a means to induce oral tolerance in 6 patients with early stage PBC, and to determine what, if any, clinical improvement was manifest by the induction of oral tolerance. After 6-month of oral antigen administration, we did not detect any encouraging changes in the titers of AMA. We also did not find any improvement in liver biochemistries. During the subsequent 6 months, no apparent changes were noted, either in the titers of AMA or in liver biochemistries. The results of this trial were thus discouraging.

In the early phase of this study, 1 of 6 patients had to discontinue study medication because of the deterioration in a pre-existing skin rash after the medication was begun. This lesion disappeared within 2 weeks after stopping medication. Another patient, who presented with lymphadenopathy and Raynaud's phenomenon at entry, noticed mild deterioration of these manifestations during the administration of study medication and improvement to the pre-existed level after completing 6 months of

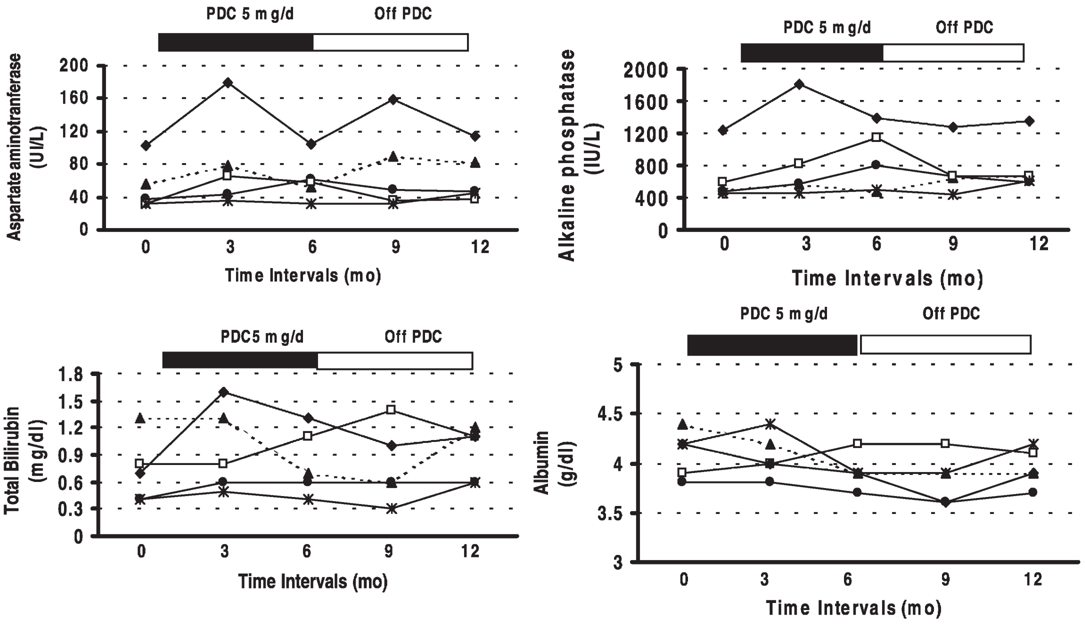

FIGURE 2 Serum alkaline phosphatase, aspartate aminotransferase, total bilirubin, and albumin in patients before and after medication. (a) Alkaline phosphatase as U/l (normal range: 81–213 U/l). (b) Aspartate aminotransferase as U/l (normal range: 12–31 U/l). (c) Total bilirubin as mg/dl (normal range: 0.1–1.1 mg/dl). (d) Albumin as g/dl (normal range: 3.5–5.0 g/dl). The horizontal axis of each chart is the time interval of 12 months study duration. The dotted line with open diamonds represents patient No. 1. The solid line with closed squares represents patient No. 2. The dotted line with stars represents patient No. 3. The solid line with open squares represents patient No. 4. The solid line with closed triangles represents patient No. 5.

medication. It is not certain whether there was any causal relationship between this medication and these manifestations. Otherwise, the medication was tolerable without any reports of severe adverse effect during the duration of the study. Many histopathological and immunological findings suggest an important role of cellular immunity in the pathogenesis of PBC (Jones *et al.*, 1995; Martinez *et al.*, 1995; Ohmoto *et al.*, 1997). Considering the complexity of the immunological mechanisms in PBC, we may need to evaluate the change of autoimmunity from different perspectives to confirm whether oral tolerance was induced, such as testing mucosal T cells and anti-inflammatory cytokines in addition to peripheral T cell profiles and autoantibodies.

In this study, we adopted several humoral immunological markers to evaluate the induction of oral tolerance. It is interesting that patient No. 5, who had the shortest interval from initial diagnosis, showed the greatest decrease in autoantibody titers during the study. Moreover, plasma cells producing autoantibodies are long-lived and therefore, a change in autoantibody titers may occur slowly over time.

With respect to liver biochemistries, we could not see any improvement during the study duration. Regardless of whether oral tolerance was successfully induced or not, it is obvious that the current medication was not clinically effective. Even in the patients with a slight decrease in AMA titers during the study, we could not see any response in the liver biochemistries. Whether oral tolerance was successfully induced, or whether any humoral response seen during this study was not enough to drive abnormal autoimmunity to normal, cannot be answered by this study. There is also the question of whether some patients with autoimmune disease are incapable of generating tolerance to orally administered antigens. Also it is still unclear whether the induction of oral tolerance is beneficial in the treatment of patients with PBC.

Despite the data in this study, the induction of oral tolerance could be a novel, safe, and attractive therapeutic concept in PBC. Oral tolerance therapy has several benefits including rare adverse effects, easy application, and no requirement for long-term medication. Considering that most current treatments of PBC require lifelong medication, it would be of great benefit to the patients if we could provide a medication-free or less medicated life. This approach has been tried in several other autoimmune diseases such as multiple sclerosis, allergic uveitis, rheumatoid arthritis, and myasthenia gravis and has occasionally shown encouraging clinical data especially in rheumatoid arthritis (Trentham *et al.*, 1993) and multiple sclerosis (Weiner *et al.*, 1993). However, there are still challenges in the realization of oral tolerance therapy in human autoimmune disease, which include determination of the optimal dosage, molecular configuration of the antigen, delivery system, and possible variance derived from individual backgrounds such as age, gender, genetic predisposition, and also gut flora.

Different amounts of antigen induce different mechanisms of immune tolerance. Low doses of oral antigen induce transferable suppressor T cell mediated tolerance (Chen *et al.*, 1994), and in contrast, high dose oral antigen induces clonal energy/deletion (Whitacre *et al.*, 1991; Chen *et al.*, 1995). We adopted a high dose of oral antigen administration in this study because it seems to be more suitable for the long-term maintenance of the orally tolerant state. We administered 5 mg of PDC during a fasting period. Although the amount we used was 10–100 times more than the estimated amount in regular diets, the ingested protein is labile to degradation during gastro-intestinal transit by acid and proteases, such as trypsin, even during the fasting period and may be insufficient for inducing oral tolerance. We may need to consider antigen feeding in combination with a trypsin inhibitor and/or sodium bicarbonate in future trials, which has proved effective in the field of experimental encephalomyelitis (Whitacre *et al.*, 1996).

The gut immune system responds differentially to differences in molecular complexity and small peptide sequences. In the studies of experimental autoimmune myasthenia gravis, the oral administration of Torpedo AchR (nicotinic acetylcholine receptor), one of the xenogeneic and highly immunogenic autoantigens, during an acute phase showed induction of autoantibodies and an increase in the autoantibody titers to self muscle AchR (Drachman *et al.*, 1996; Shi *et al.*, 1998). In contrast, the recombinant fragment of human AchR alpha-subunit, which is a syngeneic, modified, less immunogenic autoantigen, showed active suppression via Th2/Th3-secreted regulatory cytokines and induced tolerance when it was orally administered in experimental autoimmune myasthenia gravis (Im *et al.*, 1999). We used a bovine extract containing intact PDC, and some BCKD and OGDC. Animal studies have shown that intact bovine PDC with multi-subunit quartenary structure, but not the monomeric recombinant PDC-E2 inner lipoyl domain, could cause an immunological response and produce AMA (Jones *et al.*, 1999). Considering the results from this previous report, intact bovine PDC may have high immunogenicity in humans compared to monomeric recombinant PDC-E2, despite high homology of PDC molecules among species. This may provide a partial explanation about the increase in the titers of autoanti-bodies for some patients, and possible deterioration of pre-existing manifestations seen in 2 patients during medication administration. Given the effect of molecular differences on tolerance induction in experimental myasthenia gravis, and the breakdown of tolerance in experimental autoimmune cholangitis, using monomeric PDC-E2 may be another option for inducing higher oral tolerance.

### References

Angulo, P., Lindor, K.D., Therneau, T.M., Jorgensen, R.A., Malinchoc, M., Kamath, P.S. and Dickson, E.R. (1999) "Utilization of the Mayo risk score in patients with primary biliary cirrhosis receiving ursodeoxycholic acid", *Liver* **19**, 115–121.

Angulo, P., Jorgensen, R.A., Keach, J.C., Dickson, E.R., Smith, C. and Lindor, K.D. (2000a) "Oral budesonide in the treatment of patients with primary biliary cirrhosis with a suboptimal response to ursodeoxycholic acid", *Hepatology* **31**, 318–323.

Angulo, P., Patel, T., Jorgensen, R.A., Therneau, T.M. and Lindor, K.D. (2000b) "Silymarin in the treatment of patients with primary biliary cirrhosis with a suboptimal response to ursodeoxycholic acid", *Hepatology* **32**, 897–900.

Bitar, D.M. and Whitacre, C.C. (1988) "Suppression of experimental autoimmune encephalomyelitis by the oral administration of myelin basic protein", *Cell Immunol.* **112**, 364–370.

Chen, Y., Kuchroo, V.K., Inobe, J., Hafler, D.A. and Weiner, H.L. (1994) "Regulatory T cell clones induced by oral tolerance: suppression of autoimmune encephalomyelitis", *Science* **265**, 1237–1240.

Chen, Y., Inobe, J., Marks, R., Gonnella, P., Kuchroo, V.K. and Weiner, H.L. (1995) "Peripheral deletion of antigen-reactive T cells in oral tolerance", *Nature* **376**, 177–180.

Drachman, D.B., Okumura, S., Adams, R.N. and McIntosh, K.R. (1996) "Oral tolerance in myasthenia gravis", *Ann. N. Y. Acad. Sci.* **778**, 258–272.

Gershwin, M.E., Mackay, I.R., Sturgess, A. and Coppel, R.L. (1987) "Identification and specificity of a cDNA encoding the 70 kd mitochondrial antigen recognized in primary biliary cirrhosis", *J. Immunol.* **138**, 3525–3531.

Heathcote, J., Ross, A. and Sherlock, S. (1976) "A prospective controlled trial of azathioprine in primary biliary cirrhosis", *Gastroenterology* **70**, 656–660.

Im, S.H., Barchan, D., Fuchs, S. and Souroujon, M.C. (1999) "Suppression of ongoing experimental myasthenia by oral treatment with an acetylcholine receptor recombinant fragment", *J. Clin. Investig.* **104**, 1723–1730.

Jones, D., Palmer, J., Yeaman, S., Kirby, J. and Bassendine, M. (1999) "Breakdown of tolerance to pyruvate dehydrogenase complex in experimental autoimmune cholangitis: a mouse model of primary biliary cirrhosis", *Hepatology* **30**, 65–70.

Jones, D.E., Palmer, J.M., James, O.F., Yeaman, S.J., Bassendine, M.F. and Diamond, A.G. (1995) "T-cell responses to the components of pyruvate dehydrogenase complex in primary biliary cirrhosis", *Hepatology* **21**, 995–1002.

Jones, D.E., Palmer, J.M., Kirby, J.A., De Cruz, D.J., McCaughan, G.W., Sedgwick, J.D., Yeaman, S.J., Burt, A.D. and Bassendine, M.F. (2000) "Experimental autoimmune cholangitis: a mouse model of immune-mediated cholangiopathy", *Liver* **20**, 351–356.

Kaplan, M.M. and Knox, T.A. (1991) "Treatment of primary biliary cirrhosis with low-dose weekly methotrexate", *Gastroenterology* **101**, 1332–1338.

Leung, P.S., Krams, S., Munoz, S., Surh, C.P., Ansari, A., Kenny, T., Robbins, D.L., Fung, J., Starzl, T.E., Maddrey, W., Coppel, R.L. and Gershwin, M.E. (1992) "Characterization and epitope mapping of human monoclonal antibodies to PDC-E2, the immunodominant autoantigen of primary biliary cirrhosis", *J. Autoimmun.* **5**, 703–718.

Leung, P.S.C., Coppel, R.L., Ansari, A., Munoz, S. and Gershwin, M.E. (1997) "Antimitochondrial antibodies in primary biliary cirrhosis", *Semin. Liver Dis.* **17**, 61–69.

Lindor, K.D., Dickson, E.R., Baldus, W.P., Jorgensen, R.A., Ludwig, J., Murtaugh, P.A., Harrison, J.M., Wiesner, R.H., Anderson, M.L., Lange, S.M., *et al.* (1994) "Ursodeoxycholic acid in the treatment of primary biliary cirrhosis", *Gastroenterology* **106**, 1284–1290.

Lindor, K.D., Dickson, E.R., Jorgensen, R.A., Anderson, M.L., Wiesner, R.H., Gores, G.J., Lange, S.M., Rossi, S.S., Hofmann, A.F. and Baldus, W.P. (1995) "The combination of ursodeoxycholic acid and methotrexate for patients with primary biliary cirrhosis: the results of a pilot study", *Hepatology* **22**, 1158–1162.

Lindor, K.D., Therneau, T.M., Jorgensen, R.A., Malinchoc, M. and Dickson, E.R. (1996) "Effects of ursodeoxycholic acid on survival in patients with primary biliary cirrhosis", *Gastroenterology* **110**, 1515–1518.

Lindor, K.D., Jorgensen, R.A., Therneau, T.M., Malinchoc, M. and Dickson, E.R. (1997) "Ursodeoxycholic acid delays the onset of esophageal varices in primary biliary cirrhosis", *Mayo Clin. Proc.* **72**, 1137–1140.

Lombard, M., Portmann, B., Neuberger, J., Williams, R., Tygstrup, N., Ranek, L., Ring-Larsen, H., Rodes, J., Navasa, M., Trepo, C., *et al.* (1993) "Cyclosporin A treatment in primary biliary cirrhosis: results of a long-term placebo controlled trial [see comments]", *Gastro-enterology* **104**, 519–526.

Martinez, O., Villanueva, J., Gershwin, M.E. and Krams, S. (1995) "Cytokine patterns and cytotoxic mediators in primary biliary cirrhosis", *Hepatology* **21**, 113–119.

Mitchison, H.C., Bassendine, M.F., Malcolm, A.J., Watson, A.J., Record, C.O. and James, O.F. (1989) "A pilot, double-blind, controlled 1-year trial of prednisolone treatment in primary biliary cirrhosis: hepatic improvement but greater bone loss", *Hepatology* **10**, 420–429.

Nagler-Anderson, C., Bober, L.A., Robinson, M.E., Siskind, G.W. and Thorbecke, G.J. (1986) "Suppression of type II collagen-induced arthritis by intragastric administration of soluble type II collagen", *Proc. Natl Acad. Sci. USA* **83**, 7443–7446.

Nakanuma, Y., Tsuneyama, K., Kono, N., Hoso, M., Van de Water, J. and Gershwin, M.E. (1995) "Biliary epithelial expression of pyruvate dehydrogenase complex in primary biliary cirrhosis: an immunohistochemical and immunoelectron microscopic study", *Hum. Pathol.* **26**, 92–98.

Nussenblatt, R.B., Caspi, R.R., Mahdi, R., Chan, C.C., Roberge, F., Lider, O. and Weiner, H.L. (1990) "Inhibition of S-antigen induced experimental autoimmune uveoretinitis by oral induction of tolerance with S-antigen", *J. Immunol.* **144**, 1689–1695.

Ohmoto, M., Yamamoto, K., Nagano, T., Matsumoto, S., Kobashi, H., Okamoto, R. and Tsuji, T. (1997) "Accumulation of multiple T-cell clonotypes in the liver of primary biliary cirrhosis", *Hepatology* **25**, 33–37.

Peterson, K.E. and Braley-Mullen, H. (1995) "Suppression of murine experimental autoimmune thyroiditis by oral administration of porcine thyroglobulin", *Cell Immunol.* **166**, 123–130.

Poupon, R.E., Lindor, K.D., Cauch-Dudek, K., Dickson, E.R., Poupon, R. and Heathcote, E.J. (1997) "Combined analysis of randomized controlled trials of ursodeoxycholic acid in primary biliary cirrhosis", *Gastroenterology* **113**, 884–890.

Shi, F.D., Bai, X.F., Li, H.L., Huang, Y.M., Van der Meide, P.H. and Link, H. (1998) "Nasal tolerance in experimental autoimmune myasthenia gravis (EAMG): induction of protective tolerance in primed animals", *Clin. Exp. Immunol.* **111**, 506–512.

Thompson, H.S. and Staines, N.A. (1986) "Gastric administration of type II collagen delays the onset and severity of collagen-induced arthritis in rats", *Clin. Exp. Immunol.* **64**, 581–586.

Trentham, D.E., Dynesius-Trentham, R.A., Orav, E.J., Combitchi, D., Lorenzo, C., Sewell, K.L., Hafler, D.A. and Weiner, H.L. (1993) "Effects of oral administration of type II collagen on rheumatoid arthritis", *Science* **261**, 1727–1730.

Tsuneyama, K., Van de water, J., Leung, P.S.C., Cha, S., Nakanuma, Y., Kaplan, M., De Lellis, R., Coppel, R., Ansari, A. and Gershwin, M.E. (1995) "Abnormal expression of the E2 component of the pyruvate dehydrogenase complex on the luminal surface of biliary epithelium occurs before major histocompatibility complex class II and BB1/B7 expression", *Hepatology* **21**, 1031–1037.

Van de Water, J., Gershwin, M.E., Leung, P., Ansari, A. and Coppel, R.L. (1988) "The autoepitope of the 74-kD mitochondrial autoantigen of primary biliary cirrhosis corresponds to the functional site of dihydrolipoamide acetyltransferase", *J. Exp. Med.* **167**, 1791–1799.

Wang, Z.Y., Qiao, J. and Link, H. (1993) "Suppression of experimental autoimmune myasthenia gravis by oral administration of acetylcholine receptor", *J. Neuroimmunol.* **44**, 209–214.

Weiner, H.L., Mackin, G.A., Matsui, M., Orav, E.J., Khoury, S.J., Dawson, D.M. and Hafler, D.A. (1993) "Double-blind pilot trial of oral tolerization with myelin antigens in multiple sclerosis", *Science* **259**, 1321–1324.

Whitacre, C.C., Gienapp, I.E., Orosz, C.G. and Bitar, D.M. (1991) "Oral tolerance in experimental autoimmune encephalomyelitis III. Evidence for clonal anergy", *J. Immunol.* **147**, 2155–2163.

Whitacre, C.C., Gienapp, I.E., Meyer, A., Cox, K.L. and Javed, N. (1996) "Treatment of autoimmune disease by oral tolerance to autoantigens", *Clin. Immunol. Immunopathol.* **80**, S31–S39.

Wiesner, R.H., Ludwig, J., Lindor, K.D., Jorgensen, R.A., Baldus, W.P., Homburger, H.A. and Dickson, E.R. (1990) "A controlled trial of cyclosporine in the treatment of primary biliary cirrhosis [see comments]", *N. Engl. J. Med.* **322**, 1419–1424.

Yeaman, S.J., Fussey, S.P., Danner, D.J., James, O.F., Mutimer, D.J. and Bassendine, M.F. (1988) "Primary biliary cirrhosis: identification of two major M2 mitochondrial autoantigens", *Lancet* **1**, 1067–1070.

Yeaman, S.J. (1989) "The 2-oxo acid dehydrogenase complexes: recent advances", *Biochem. J.* **257**, 625–632.

